# Sustained intraocular pressure lowering from suprachoroidal injection of a latanoprost lipid nanoparticle delivery system in the rabbit

**DOI:** 10.3389/fmed.2026.1776626

**Published:** 2026-03-06

**Authors:** Phey Feng Lo, Sai Bo Bo Tun, Ryan E. K. Man, Joanna Marie Fianza Busoy, Shamira Perera, Veluchamy A. Barathi, Tina T. Wong

**Affiliations:** 1 Singapore Eye Research Institute, Singapore, Singapore; 2 Singapore National Eye Centre, Singapore, Singapore; 3 Duke-NUS Medical School, Singapore, Singapore

**Keywords:** animal model, glaucoma, liposomal latanoprost, nanoparticle drug delivery system, suprachoroidal space, sustained drug delivery

## Abstract

**Background:**

Current treatments for glaucoma to lower intraocular pressure (IOP) are dependent on patient adherence and bioavailability after bypassing ocular barriers. Our study investigated the safety and efficacy of a liposomal nanocarrier for sustained release of latanoprost injected into the suprachoroidal space in the rabbit eye.

**Methods:**

Dutch-Belt rabbits were injected with a single dose of 150 μL of 2 mg/mL of liposomal latanoprost formulation into the suprachoroidal space. The eyes were clinically monitored for adverse events and the IOP recorded.

**Results:**

IOP reduced from baseline to 8 h post liposomal latanoprost injection by 28% (5.1 ± 2.12 mmHg), reaching a maximum of 40% (7.2 ± 1.9 mmHg) at Day 5. Low IOP was sustained until Day 56 returning to baseline at Day 90. Safety assessment of the eyes showed no adverse events and good tolerability of the drug.

**Conclusion:**

A single dose of liposomal latanoprost into the suprachoroidal space can lower the IOP for up to 56 days making it a promising therapeutic agent for sustained delivery drugs to the suprachoroidal space.

## Introduction

Glaucoma, a chronic and progressive optic neuropathy, affects 80 million people worldwide, and this number continues to rise (1). Despite being the leading cause of irreversible blindness worldwide, intraocular pressure (IOP) remains the only modifiable risk factor identified. Eye drops are the primary treatment for IOP lowering however, patient compliance is poor. A study of persistence rate of patients using single IOP lowering drops after one year was 22.5, and 11.5% after three years (2). A single dose of latanoprost, a well-known prostaglandin, reduces IOP by 25–35%, which is considered a clinically therapeutic amount. To achieve this, current dosing regimens require at least one drop to be instilled every 24 h (3). Less than 5% of a topical eyedrop penetrates the cornea, the remaining 95% is lost through lacrimal clearance, blink reflex, nasolacrimal drainage or other ocular barriers (4). Sustained drug delivery allows for the maintenance of optimal therapeutic concentrations in the eye, increasing drug permeation and bioavailability to targeted ocular tissues, such as the ciliary body and uveoscleral outflow (5, 6). It also obviates the need for preservatives and reduces extraocular side effects of the drug, which can significantly impact patient compliance.

To optimise the efficacy of sustained-release drugs, we need to further explore ideal sites for targeted delivery of these therapeutic agents. The suprachoroidal space bypasses ocular barriers for drug delivery, as it expands as a distensible and compartmentalised space (7). The use of this unique space has been investigated by medical retina specialists for the treatment of macular oedema (8). Clinical trials of suprachoroidal injections of triamcinolone in retinal disease have demonstrated safety and efficacy (8). In glaucoma, the suprachoroidal space is considered an optimal site for sustained drug delivery. The main challenge lies in maximising drug dosing to ensure optimal efficacy as well as ensuring the drug is consistently delivered to the correct anatomical space.

The purpose of this study is to investigate the suprachoroidal route for sustained drug delivery in glaucoma. We investigated the IOP lowering effect following the injection of liposomal latanoprost (LipoLAT®) into the suprachoroidal space in Dutch-Belt rabbits and monitored safety in this route of administration.

## Materials and methods

**Figure 1:**
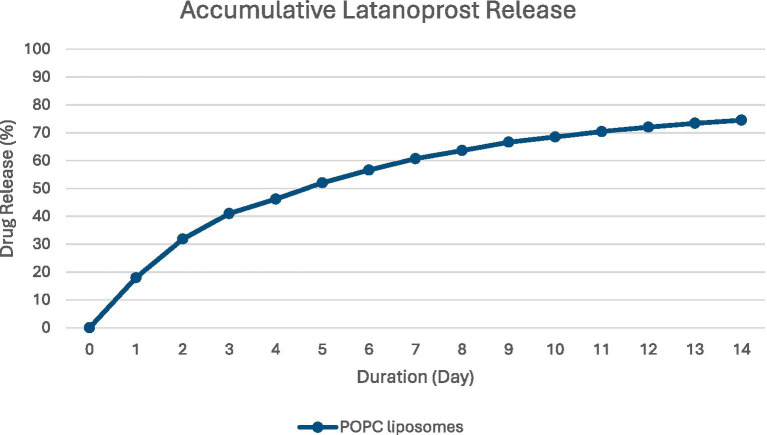
Accumulative release of POPC-latanoprost over time.

POLAT-001 (Peregrine Ophthalmic, Singapore) is a nanoliposomal latanoprost formulation with a high loading of drug at 2 mg/mL. The liposome used for making LipoLAT is 1-palmitoyl-2-oleoyl-sn-glycero-3-phosphocholine (POPC) (16:0/18:1). The vesicle size of LipoLAT is between 50 and 150 nm with a drug to lipid ratio of approximately 0.1. Figure 1 shows the accumulative *in vitro* release profile of liposomal latanoprost. Approximately 20% of the drug is released within the first 24-h and 47% of the drug released within the first 4-day period. The accumulative released latanoprost 75% after 14 days of incubation in Phosphate Buffered Saline (PBS) maintained at 37 °C.

The suprachoroidal injector (SupraMedical, Israel) with a 30-gauge needle, attached to an extension tubing and a 1 mL syringe, was used to deliver the formulation. The length of the injector needle was adjusted according to the calibration table from the manufacturer by inputting readings of scleral thickness combined with a correction factor obtained by ultrasound biomicroscopy (UBM). After the dial was set in position, the injector was placed at the desired location to penetrate the suprachoroidal space. The injection was delivered over 5–10 s by slowly pressing the syringe plunger. After the drug was delivered, the plunger was released, and the injector gradually removed.

### Surgical technique

Dutch-Belt rabbits were used in this study. Rabbits were divided into two groups. Group A (*n* = 10 eyes) received one suprachoroidal injection of 150 μL liposomal latanoprost, whilst Group B (*n* = 6 eyes) received a suprachoroidal injection of a blank liposomal nanocarrier of the same volume. Both groups received their injections at similar time points at 09:00. A total of 8 Dutch-Belt rabbits were used for both groups described, and both eyes of the rabbits were included in the study.

All procedures were performed under the microscope by a single trained surgeon. The rabbits were anaesthetised with an intramuscular injection (Ketamine 50 mg/kg body weight, Xylazine at 10 mg/kg body weight) and received 1–2 drops of topical anaesthesia (0.5% Propacaine Hydrochloride). The study eye was cleaned with 5% Povidone Iodine followed by a saline wash. The injector was placed at a 2 or 6 o’clock position at the limbus, avoiding the extraocular muscles. Injection was performed 3–4 mm posterior to the limbus. The base of the injector was anchored on the sclera, the injector needle was extended into the suprachoroidal space, and 150 μL of liposomal latanoprost or blank liposomal nanocarrier was injected into the space. The needle was withdrawn a few seconds later to minimise reflux of the injected drug. An OCT was performed immediately after the procedure to ensure the drug was delivered into the correct space. This was confirmed by observing the distension of the suprachoroidal space post-injection. The operated eye was given a stat dose of topical tobramycin 3 mg/mL (Tobrex®, Novartis). All procedures were done according to protocols approved by SingHealth Institutional Animal Care and Use Committee (IACUC; AAALAC accredited) 2021/SHS/1630, and in accordance with the Association for Research in Vision and Ophthalmology (ARVO) Statement for the Use of Animals in Ophthalmic and Visual Research.

### IOP measurement on rabbits

IOPs were measured using a calibrated Tonopen XL® (Reichert Ophthalmic Instruments, Depew, NY). Topical anaesthesia was applied before IOP measurements. Animals were handled with care by trained research veterinarians to ensure stable and consistent IOP readings. Each rabbit was acclimatised to the IOP measurement procedure for at least 7 days to obtain a stable baseline IOP. On the day of the procedure, IOPs were recorded at 1 h, 4 h, 8 h, and 24 h post-procedure. Three readings were taken from each eye, and an average value was obtained. IOP measurements were then taken daily for 14 days, followed by weekly for 6 weeks, then biweekly until IOP returned to baseline levels.

### Clinical evaluation, slit lamp biomicroscopy, anterior segment imaging

Rabbits receiving a suprachoroidal injection had their scleral thickness measured with Optovue RTVue Optical Coherence Tomography (OCT). The sclera thickness at 3 mm behind the corneoscleral junction was measured. An average of three readings were taken to determine the mean scleral thickness. The length of the injector needle was subsequently calibrated by adding 300 μm to the mean scleral thickness. This additional value was based on the calibration recommended by the manufacturer to ensure that the drug was delivered into the suprachoroidal space.

Slit lamp biomicroscopy of the external eye and anterior chamber was performed before the injections and at pre-determined timepoints. Anterior segment photographs, anterior segment optical coherence tomography (AS-OCT) (RTVue, FD-OCT; Optovue Inc., Fremont CA), and OCT of macula (HRT Spectralis-OCT, Heidelberg, Germany) were performed pre-injection, and post-injection at weeks 1, 4, 8 and 12. Visual inspection of the eyes and the adnexal tissues for signs of latanoprost’s side effects were conducted daily. Rabbits were also monitored for any gross changes such as discharge, squinting or general behaviour suggesting pain or severe discomfort. A modified Hackett-Mcdonald Scoring Scale was used to grade conjunctival hyperaemia/ erythema, corneal vascularisation, anterior and posterior cells (Tables 1 and 2). Scores obtained were then averaged per animal.

**Table 1:** Scoring system for assessment of conjunctival congestion (hyperaemia).

Score	Description
0	Normal. May appear blanched to reddish pink without perilimbal injection (except at 12:00 and 6:00 positions) with vessels of the palpebral and bulbar conjunctiva easily observed.
1+	A flushed reddish colour predominantly confined to the bulbar conjunctiva with some perilimbal injection. Primarily confined to the lower and upper parts of the eye from the 4:00 and 7:00 o’clock and the 11:00 and 1:00 o’clock positions.
2+	Bright red colour of the bulbar and palpebral conjunctiva with accompanying perilimbal injection covering at least 75% of the circumference of the perilimbal region.
3+	Dark, beefy red colour with congestion of the bulbar and the palpebral conjunctiva along with pronounced perilimbal injection. Petechia may be present on the conjunctiva. The petechiae generally predominate along the nictitating membrane and the upper palpebral conjunctiva.

The degree of pigmentation in eyes may preclude accurate scoring of this parameter.

**Table 2:** Scoring system for anterior and posterior cells.

Score	Description
0	No cells observed
0.5+	1 to 5 cells per single field of focused beam
1+	5 to 25 cells per single field of focused beam
2+	25 to 50 cells per single field of focused beam
3+	50 to 100 cells per single field of focused beam
4+	>100 cells per single field of focused beam

## Statistical analysis

Statistical analysis was done using GraphPad Prism (Version 10.2.0) and STATA 17.0 (Statacorp, TX, USA). Mean and standard deviation (SD) were used for continuous variables, whilst percentages were used for categorical variables. For comparison of differences in mean reduction (%) in IOP between both groups, mixed models to account for a potential clustering effect between both eyes were used to estimate the within- and between-group differences in the liposomal latanoprost and blank liposomal groups. Q-Q plots showed non-normal distributions within both groups (data not shown); as such, robust standard errors were utilised to account for these non-normal data. Lastly, a non-parametric Wilcoxon signed-rank test was used to assess the changes in retinal thickness over time monocularly in both the treatment and control groups. A *p*-value of <0.05 was considered statistically significant.

## Results

### Intraocular pressure

The average baseline IOP was 15.61 ± 3.76 mmHg in all eyes (*n* = 16). A lower baseline IOP in the blank liposome group was noted, 11.58 mmHg compared with 18.03 mmHg in the liposomal latanoprost group. Mixed model calculations within group changes showed a 5.1 mmHg (28.3%) drop in IOP from baseline to 8 h post-liposomal latanoprost injection, reaching a maximum of 7.2 mmHg (40%) decrease at Day 7 (Table 3). This IOP lowering effect of at least 20% was sustained until Day 56 before observing a gradual return to baseline IOP at Day 90. In comparison, the eyes that were administered with blank liposomes recorded a maximum IOP lowering of 0.9 mmHg (7.7%) at day 7 (Table 3). IOP gradually returned to baseline by day 28 and remained stable with minimal fluctuations throughout the study period. Comparison of group differences over tine showed a significant IOP decrease in the liposomal latanoprost injection group at 8 h, days 1, 7, 28, and 56 compared to the blank liposomal injection (Table 4).

**Table 3:** Comparison of within group changes in intraocular pressure over time.

	Liposomal latanoprost		Blank liposome	
	Mean difference in mmHg^a^ (95% CI)	*p* value	Mean difference in mmHg^a^ (95% CI)	*p* value
Baseline	Reference	–	Reference	–
8 h	−5.10 (−6.85 to −3.35)	<0.001	0.36 (0.00 to 0.72)	0.047
Day 1	−6.00 (−8.08 to −3.92)	<0.001	−0.61 (−1.15 to −0.07)	0.025
Day 7	−7.20 (−7.83 to −6.57)	<0.001	−0.89 (−2.66 to 0.88)	0.325
Day 28	−5.13 (−5.84 to −4.43)	<0.001	−0.28 (−0.67 to 0.11)	0.166
Day 56	−4.67 (−5.67 to −3.66)	<0.001	−0.53 (−1.37 to 0.32)	0.221
Day 84	−2.27 (−3.31 to −1.22)	<0.001	−0.61 (−1.32 to 0.10)	0.091
Day 91	−0.59 (−1.67 to 0.49)	0.283	–	–

a Estimated with mixed models adjusted for within-eye correlation and using robust standard errors. CI: confidence interval.

**Table 4:** Comparison of between group differences in intraocular pressure over time between rabbit latanoprost and blank liposomal groups.

	Mean difference in mmHg^a^ Controls—Cases (95% CI)	*p* value
Baseline	0 (0)	–
8 h	5.46 (3.76 to 7.16)	<0.001
Day 1	5.39 (3.34 to 7.44)	<0.001
Day 7	6.31 (4.65 to 7.97)	<0.001
Day 28	4.86 (4.10 to 5.61)	<0.001
Day 56	4.14 (2.93 to 5.35)	<0.001
Day 84	1.65 (0.48 to 2.83)	0.006

a Estimated with mixed models adjusted for within-eye correlation and using robust standard errors. CI: confidence interval.

### Anterior segment optical coherence tomography (AS-OCT)

**Figure 2:**
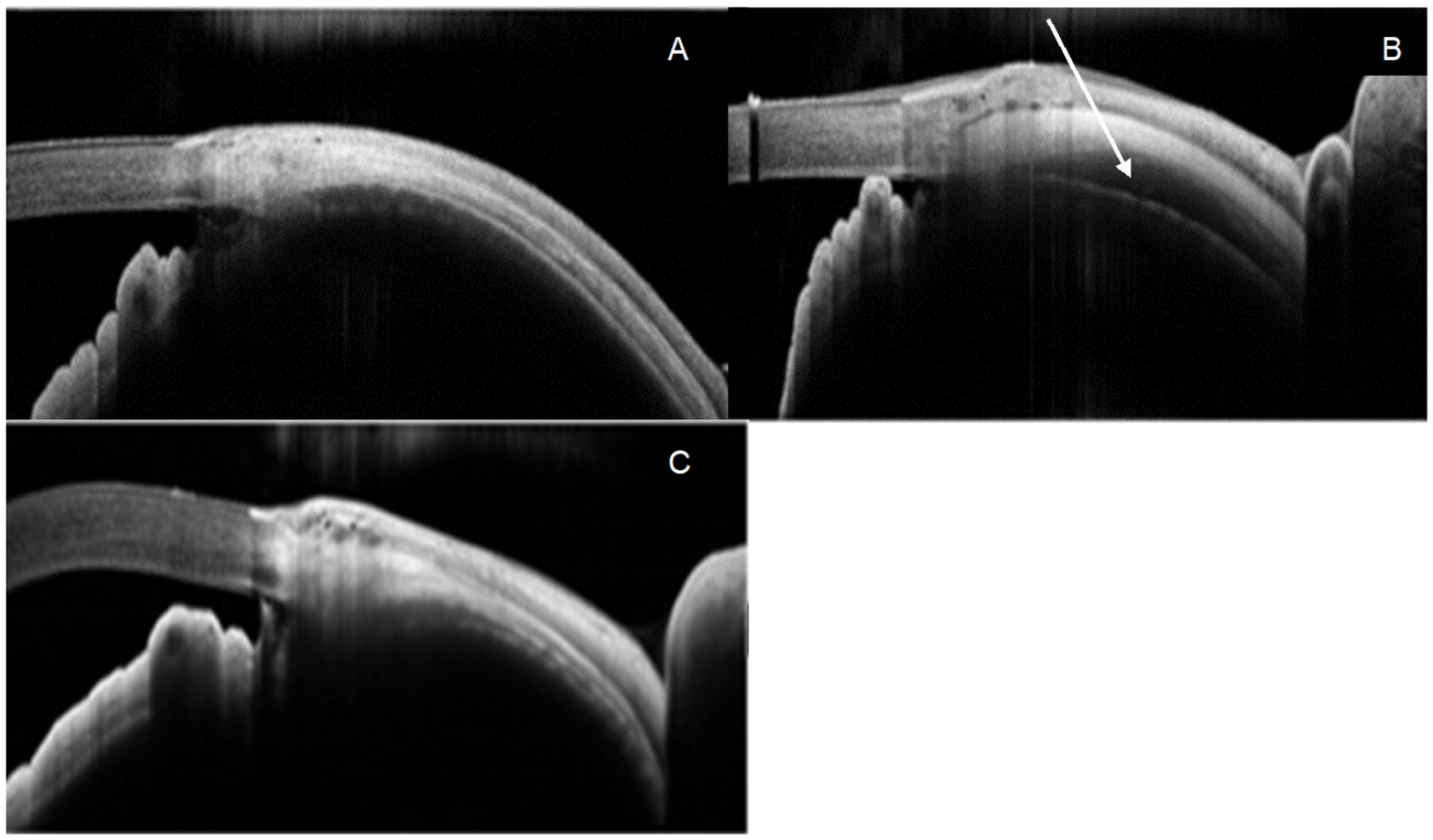
Anterior segment optical coherence tomography **(A)** pre-injection and **(B)** post-injection confirming injection of liposomal latanoprost into the suprachoroidal space as shown by the arrow. **(C)** AS OCT 1 month post-injection demonstrating obliteration of the suprachoroidal space and reattachment of the ciliary body.

AS-OCT scans were performed on all eyes pre- and post-injections, at week 1, 4, 8, and 12. The post-injection scans were done to ensure the drug was delivered into the correct anatomical plane of the suprachoroidal space as demonstrated in Figure 2. This was consistently shown throughout all injections receiving liposomal latanoprost and blank carrier. Further AS-OCT at weeks 4, 8, and 12 indicated that the suprachoroidal space was no longer distended, indicating that the drug injected has been redistributed. This also indicated that further IOP lowering was due to the drug injected and not due to the anatomical opening of the suprachoroidal space.

### Safety data

**Figure 3:**
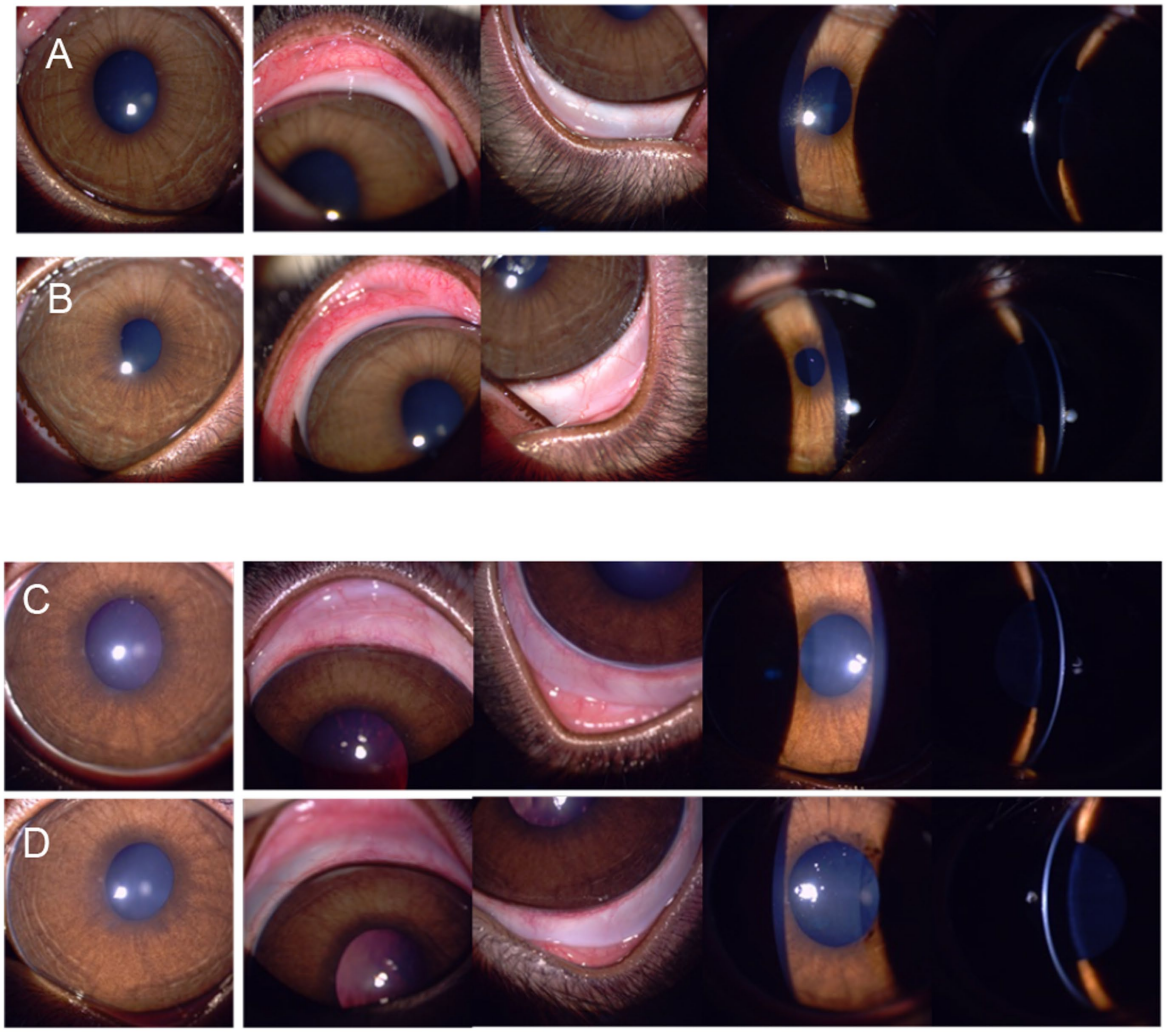
Visual inspection was done to look for signs of irritation, inflammation, or infection. The modified Hackett-Mcdonald Scoring Scale was used. **(A)** and **(B)** showed slit lamp photographs taken at baseline. **(C)** and **(D)** showed slit lamp photographs taken at week 12 post-suprachoroidal liposomal latanoprost injection.

All animals in all groups underwent ophthalmic examinations pre- and post-injection. Daily cage-side observations were done twice a day during the experimental period. All rabbits were physically restrained for clinical examinations. Visual inspection of signs of conjunctival irritation, ocular inflammation or infection was done daily in all eyes post-injection. As shown in Figure 3, in both groups, the conjunctiva was white and quiet, the cornea clear, and there was no evidence of hyperaemia, inflammation, or infection at the site of injection or its surrounding area. Slit lamp biomicroscopy was used for clinical scoring of aqueous flare and cells (Table 2) whilst slit lamp photographs were used to assess scoring for conjunctival hyperaemia (Table 1), corneal opacity and corneal neovascularisation. The score throughout all groups pre- and post-injection was 0.

**Figure 4:**
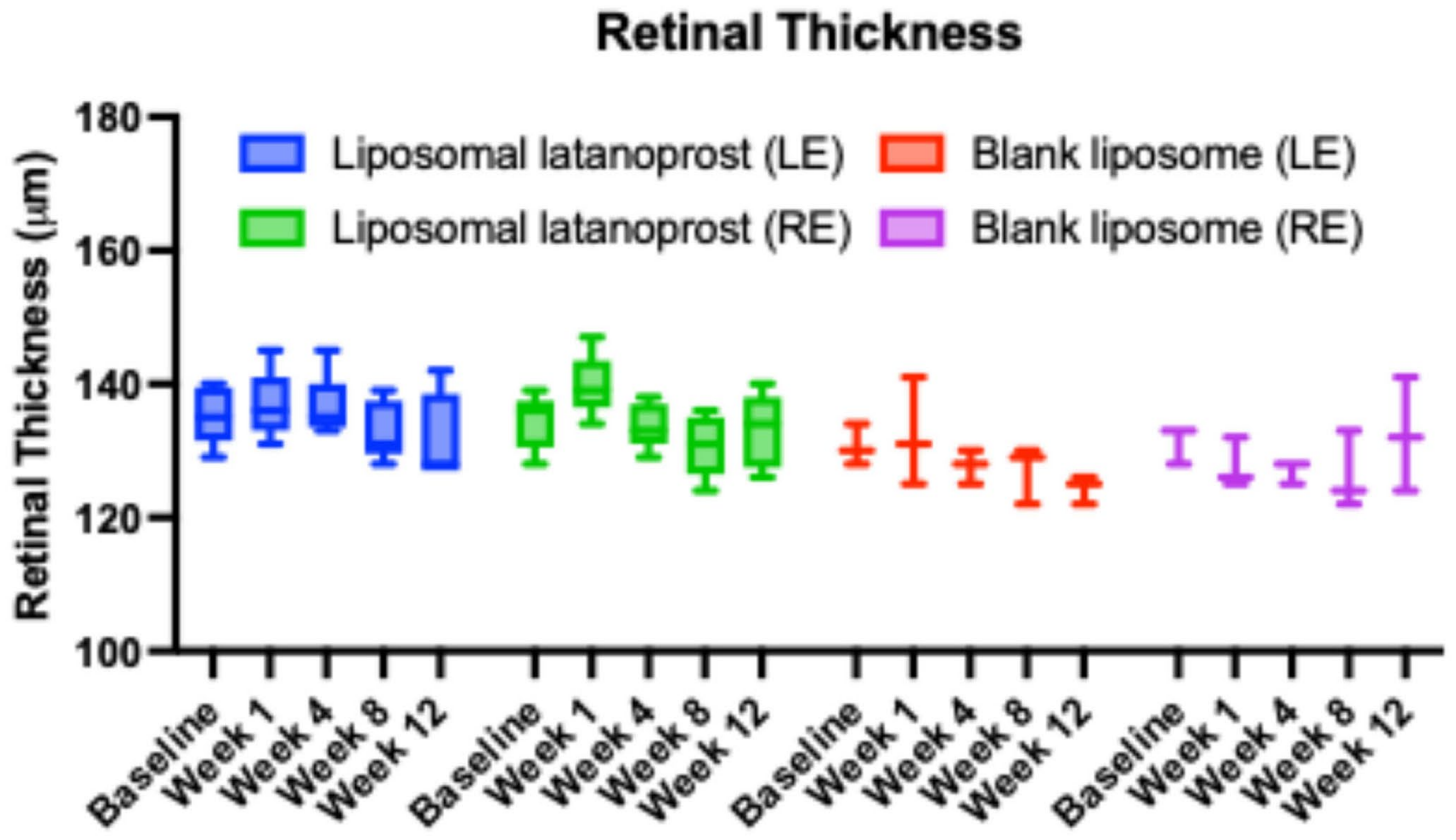
OCT retinal thickness over 90 days. The box and whisker plots showed median, interquartile range, minimum and maximum values of the retinal thickness (μm) of all groups. *p* values were calculated against the baseline retinal thickness of each group, respectively, no significant difference was found.

The OCT scans of the macula were captured at baseline, weeks 1, 4, 8, and 12. Retinal thickness remained constant from baseline to week 12 in both eyes for all treatment groups, as shown in Figure 4. The *p*-value for the difference between baseline and follow-up values was calculated using a non-parametric Wilcoxon signed-rank test and showed no significant differences.

## Discussion

Our study demonstrated a significant decrease in IOP from baseline in the suprachoroidal group receiving liposomal latanoprost injection, and this was maintained for 56 days (*p* < 0.001). This IOP-lowering effect was significantly greater than the group receiving a blank liposomal nanocarrier. We also demonstrated that the liposomal latanoprost formulation in the suprachoroidal space was safe and well-tolerated, as there were no signs of ocular irritation or inflammation.

Currently, there are two US Food and Drug Administration (FDA) approved sustained released devices for the treatment of glaucoma: A bimatoprost implant: Durysta®, (Allergan Inc., Irvine, California, USA) designed to release bimatoprost over 3–4 months and a travoprost implant: iDose®TR; (Glaukos, California, USA) designed to release travoprost up to 3 years (9, 10). Unlike these implants, the liposomal latanoprost is implant-free and comes in an off-white formulation, which is soluble and dissolves after being absorbed. The Durysta® despite being biodegradable, has currently been approved for single application use due to the results of Phase II trial, which showed significant endothelial loss (9). In addition, its significant cost curtails its use in chronic diseases like glaucoma. The iDose® is a biocompatible titanium implant that is anchored to the trabecular meshwork in a surgical implantation procedure similar to the insertion of a trabecular bypass device like the iStent (Glaukos, California, USA). The implant has a nanoporous ethylene-vinyl acetate (EVA) membrane that controls the release of travoprost into the anterior chamber. Once depleted, the implant can be removed and replaced to allow ongoing therapeutic effects. Although it has a longer duration of action, the procedure would require an operating microscope in the operating theatre and require skills in angle surgery, limiting its usability. Both these implants have shown good reduction of off-target side effects from prostaglandin but are bound by the relatively expensive nature of their mode of delivery. Comparatively, the liposomal latanoprost injection into the suprachoroidal space is an office procedure and repeatable, making it convenient and adaptable in a busy outpatient service.

Angle closure glaucoma (ACG), although less common than OAG, is a major cause of blindness worldwide contributing to 50% of the world’s blindness due to glaucoma (11). Both the Durysta® and iDose® are licensed for use in Open Angle Glaucoma (OAG) and Ocular Hypertension (OHT) as implanting the device requires visualisation of the angle. In the case of Durysta®, a particular angle width has to be determined by OCT prior, to lessen the chances of inadvertent endothelial cell loss from the procedure or from the implant being delivered into the AC. Injecting into the suprachoroidal space, on the other hand, is not dependent on the angle of the eye. In medical retina, this has become clinically feasible after the FDA approval of CLS-TA, triamcinolone acetonide injection suspension for suprachoroidal use (XIPERE®) (12). This utilises the suprachoroidal Microinjector® (Clearside Biomedical, Alpharetta, GA), specifically designed to allow reliable, office-based procedures (13). Clinical trials have shown the safety and efficacy of suprachoroidal triamcinolone injection for use in macula oedema secondary to non-infectious uveitis (12). In our study, we have shown consistent injections into the suprachoroidal space with our injector (as described above). Our liposomal latanoprost formulation, utilising our current injector, is adaptable and applicable across all types of glaucoma.

Other studies have reported injecting hypotensive agents into the supraciliary space, targeting the ciliary body as a site of aqueous production (14). Kim et al. explored injecting viscous brimonidine and sulprostone into the supraciliary space using a hollow microneedle (15). By targeting the anterior suprachoroidal space, it increases bioavailability by increasing the concentration of the drug at the site of pharmacological action (13). Despite delivering a dose 100 times lower concentration than topical delivery, a reduction up to 3 mmHg was observed for up to 9 h (15). This provided an interesting insight into injecting antiglaucoma medications into the suprachoroidal space. However, daily injections are not acceptable for patients. Subsequently, Chiang et al. reported that a single injection of brimonidine loaded microspheres into the supraciliary space demonstrated an IOP lowering of 6 mmHg, and this lasted for 35 days (16). Our study further supports this notion by demonstrating the use of the suprachoroidal space to provide sustained drug delivery for IOP lowering. Mechanically expanding the suprachoroidal space also causes IOP lowering, and this mechanism is postulated to be due to increased uveoscleral outflow or reduced aqueous humour production, similar to that of a cyclodialysis cleft, due to the injection being close to the ciliary body (17). In both groups receiving suprachoroidal injections, an initial drop was seen secondary to this effect. In the control group however, this effect was quickly reversed, and IOP returned to baseline after 24 h with minimal IOP fluctuations thereafter.

Our liposomal latanoprost product was developed by encapsulating latanoprost into a liposomal nanocarrier (average size ~100 nm). Molecular interactions between latanoprost and the liposome molecule were optimised to enable a prolonged duration of release from the nanocarrier (18). The formulation was first described in a study utilising a single subconjunctival injection of 100 μL at 1 mg/mL in rabbits and monkeys (19). This demonstrated an IOP-lowering effect for up to 50 days comparable to daily latanoprost 0.005% eye drops, with no reported toxicity issues (19). Subsequently, a similar formulation with higher loading efficiency was developed, which demonstrated a sustained IOP reduction over a timeframe of 90 days in rabbits (20). A first-in-human clinical trial provided further evidence of the safety and tolerability of this formulation as well as the extended ocular hypotensive effect of the drug (21). The subconjunctival route represents a promising strategy for sustained drug delivery. In a phase I/II study, liposomal prednisolone delivered as a subconjunctival injection post cataract surgery was shown to be a safe and effective treatment (22). However, rapid drug clearance from the subconjunctival space, due to its rich vascular and lymphatic supply remains a limitation of this route of administration (23). Liposomes have been reported to diffuse into the sclera for a limited period following subconjunctival injection, providing a more sustained drug depot, alternative delivery strategies may offer improved retention. In this context, drug delivery via the suprachoroidal route has emerged as a potential alternative for drug delivery in glaucoma.

This proof-of-concept study was primarily to determine the drug’s safety and efficacy of injection into the suprachoroidal space. However, there are several limitations in this study. Practical considerations meant that administration of the latanoprost injection was given at 9 a.m. We may have missed nocturnal readings, which arguably may cause greater IOP fluctuations; nevertheless, this allowed for regular (hourly, 4 hourly and 8 hourly) IOP readings and close observation of any adverse events. The volume of the drug injected could potentially be increased. The dosing of the liposomal latanoprost may not be optimal, and its IOP-lowering effect could be potentiated by increasing the volume delivered. The injector currently used also needs refinement as there was potential loss of drug inside the needle post-injection, resulting in a slight variability in the volume injected. Furthermore, no objective adnexal photographs were taken to look for local side effects from latanoprost. We also used normotensive rabbit eyes and demonstrated a significant IOP reduction. By applying Goldmann’s equation, we postulate that hypertensive eyes would have an even greater IOP reduction (24). Dutta et al. calculated the correlation between rabbits and humans as follows: 8 human days = 1 rabbit day (25). From this, we can extrapolate that IOP may potentially be sustained for a longer duration in humans. Moreover, a previous study conducted in non-human primates of latanoprost-loaded EggPC liposomes delivered via subconjunctival injection compared to topical latanoprost eye drops showed no statistical significance in IOP measurement between the two groups (18). This effect was sustained up to 120 days, and when a second injection was administered, the IOP was further reduced over another 180 days (18). This confirms the reproducibility and tolerability of a further injection with the liposomal formulation (18). However, further research is required to better understand these effects in hypertensive subjects and to translate these findings to humans. It is also important to note that throughout this period, there were no device-related complications causing sight-threatening complications like endophthalmitis, suprachoroidal haemorrhage, retinal detachment or any intraocular side effects like cystoid macular oedema.

In conclusion, we have demonstrated the safety and efficacy of sustained IOP lowering following liposomal latanoprost injection into the suprachoroidal space. The suprachoroidal space shows potential for greater sustained IOP lowering with a prostaglandin analogue than the subconjunctival space. Whilst further refinements are required for clinical practice, this technique is user-friendly for clinicians and adaptable in a busy outpatient setting. In addition, it improves patient care and reduces clinic burden by improving patient adherence to treatment. The cost is also competitive, making it an attractive alternative compared to the current FDA-approved sustained delivery implants. Further studies are needed to optimise dosing and improve injection techniques for better drug delivery. The absence of injection and drug-related side effects observed so far is encouraging.

## Data Availability

The original contributions presented in the study are included in the article/supplementary material, further inquiries can be directed to the corresponding author.

## References

[1] Allison K Patel D Alabi O. Epidemiology of Glaucoma: the past, present, and predictions for the future. Cureus. (2020) 12:e11686. doi: 10.7759/cureus.11686, 33391921 PMC7769798

[2] Quek DTL Ong G-T Perera SA Lamoureux EL Aung T. Persistence of patients receiving topical glaucoma monotherapy in an Asian population. Arch Ophthalmol. (2011) 129:643–8. doi: 10.1001/archophthalmol.2010.345, 21220621

[3] Larsson L. Intraocular pressure over 24 hours after single-dose administration of latanoprost 0.005% in healthy volunteers. A randomized, double-masked, placebo controlled, cross-over single center study. Acta Ophthalmol Scand. (2001) 79:567–71. doi: 10.1034/j.1600-0420.2001.790604.x, 11782220

[4] Patel A. Ocular drug delivery systems: an overview. World J Pharmacol. (2013) 2:47. doi: 10.5497/wjp.v2.i2.47, 25590022 PMC4289909

[5] Department of Ophthalmology, University Hospitals Cleveland Medical Center, Cleveland, OH 44106, USA Kesav NP Ertel MK Seibold LK Kahook MY. Sustained-release drug delivery systems for the treatment of glaucoma. Int J Ophthalmol. (2021) 14:148–59. doi: 10.18240/ijo.2021.01.21, 33469497 PMC7790669

[6] Singh RB Ichhpujani P Thakur S Jindal S. Promising therapeutic drug delivery systems for glaucoma: a comprehensive review. Ther Adv Ophthalmol. (2020) 12:251584142090574. doi: 10.1177/2515841420905740 PMC7074511 32206746

[7] Wu KY Fujioka JK Gholamian T Zaharia M Tran SD. Suprachoroidal injection: a novel approach for targeted drug delivery. Pharmaceuticals. (2023) 16:1241. doi: 10.3390/ph16091241, 37765048 PMC10535603

[8] Yeh S Khurana RN Shah M Henry CR Wang RC Kissner JM . Efficacy and safety of suprachoroidal CLS-TA for macular edema secondary to noninfectious uveitis. Ophthalmology. (2020) 127:948–55. doi: 10.1016/j.ophtha.2020.01.006, 32173113

[9] Bacharach J Tatham A Ferguson G Belalcázar S Thieme H Goodkin ML . Phase 3, randomized, 20-month study of the efficacy and safety of Bimatoprost implant in patients with open-angle Glaucoma and ocular hypertension (ARTEMIS 2). Drugs. (2021) 81:2017–33. doi: 10.1007/s40265-021-01624-9, 34724172 PMC8602154

[10] Berdahl JP Sarkisian SR Jr Ang RE Doan LV Kothe AC Usner DW . Efficacy and safety of the Travoprost intraocular implant in reducing topical IOP-lowering medication burden in patients with open-angle Glaucoma or ocular hypertension. Drugs. (2024) 84:83–97. doi: 10.1007/s40265-023-01973-7, 38060092 PMC10789685

[11] Quigley HA. The number of people with glaucoma worldwide in 2010 and 2020. Br J Ophthalmol. (2006) 90:262–7. doi: 10.1136/bjo.2005.081224, 16488940 PMC1856963

[12] Thomas J Kim L Albini T Yeh S. Triamcinolone acetonide injectable suspension for suprachoroidal use in the treatment of macular edema associated with uveitis. Expert Rev Ophthalmol. (2022) 17:165–73. doi: 10.1080/17469899.2022.2114456, 36060305 PMC9438525

[13] Kansara VS Hancock SE Muya LW Ciulla TA. Suprachoroidal delivery enables targeting, localization and durability of small molecule suspensions. J Control Release. (2022) 349:1045–51. doi: 10.1016/j.jconrel.2022.05.061, 35868358

[14] Jung JH Chae JJ Prausnitz MR. Targeting drug delivery within the suprachoroidal space. Drug Discov Today. (2019) 24:1654–9. doi: 10.1016/j.drudis.2019.03.027, 30953867 PMC6708497

[15] Kim YC Edelhauser HF Prausnitz MR. Targeted delivery of antiglaucoma drugs to the supraciliary space using microneedles. Invest Ophthalmol Vis Sci. (2014) 55:7387. doi: 10.1167/iovs.14-14651, 25212782 PMC4232008

[16] Chiang B Kim YC Doty AC Grossniklaus HE Schwendeman SP Prausnitz MR. Sustained reduction of intraocular pressure by supraciliary delivery of brimonidine-loaded poly(lactic acid) microspheres for the treatment of glaucoma. J Control Release. (2016) 228:48–57. doi: 10.1016/j.jconrel.2016.02.041, 26930266 PMC4828324

[17] Chae JJ Jung JH Zhu W Gerberich BG Fard MRB Grossniklaus HE . Drug-free, nonsurgical reduction of intraocular pressure for four months after suprachoroidal injection of hyaluronic acid hydrogel. Adv Sci. (2021) 8:2001908. doi: 10.1002/advs.202001908 PMC7816721 33511001

[18] Natarajan JV Darwitan A Barathi VA Ang M Htoon HM Boey F . Sustained drug release in nanomedicine: a long-acting Nanocarrier-based formulation for Glaucoma. ACS Nano. (2014) 8:419–29. doi: 10.1021/nn4046024, 24392729

[19] Natarajan JV Chattopadhyay S Ang M Darwitan A Foo S Zhen M . Sustained release of an anti-glaucoma drug: demonstration of efficacy of a liposomal formulation in the rabbit eye. PLoS One. (2011) 6:e24513. doi: 10.1371/journal.pone.0024513, 21931735 PMC3170360

[20] Venkatraman S Natarajan A Darwitan M Wong. Nanomedicine for glaucoma: liposomes provide sustained release of latanoprost in the eye. Int J Nanomedicine. (2012) 123:123–131. doi: 10.2147/IJN.S25468 PMC3260956 22275828

[21] Wong TT Novack GD Natarajan JV Ho CL Htoon HM Venkatraman SS. Nanomedicine for glaucoma: sustained release latanoprost offers a new therapeutic option with substantial benefits over eyedrops. Drug Deliv Transl Res. (2014) 4:303–9. doi: 10.1007/s13346-014-0196-9, 25787063

[22] Wong CW Wong E Metselaar JM Storm G Wong TT. Liposomal drug delivery system for anti-inflammatory treatment after cataract surgery: a phase I/II clinical trial. Drug Deliv Transl Res. (2022) 12:7–14. doi: 10.1007/s13346-021-00912-x, 33569720

[23] Chaw SY Novera W Chacko A-M Wong TTL Venkatraman S. In vivo fate of liposomes after subconjunctival ocular delivery. J Control Release. (2021) 329:162–74. doi: 10.1016/j.jconrel.2020.11.053, 33271203

[24] Brubaker RF. Goldmann’s equation and clinical measures of aqueous dynamics. Exp Eye Res. (2004) 78:633–7. doi: 10.1016/j.exer.2003.07.002, 15106943

[25] Dutta S Sengupta P. Rabbits and men: relating their ages. J Basic Clin Physiol Pharmacol. (2018) 29:427–35. doi: 10.1515/jbcpp-2018-0002, 29672272

